# Association Between Maternal Fluoride Exposure During Pregnancy and IQ Scores in Offspring in Canada

**DOI:** 10.1001/jamapediatrics.2019.1729

**Published:** 2019-08-19

**Authors:** Rivka Green, Bruce Lanphear, Richard Hornung, David Flora, E. Angeles Martinez-Mier, Raichel Neufeld, Pierre Ayotte, Gina Muckle, Christine Till

**Affiliations:** 1 Faculty of Health, York University, Toronto, Ontario, Canada; 2 Faculty of Health Sciences, Simon Fraser University, Burnaby, British Columbia, Canada; 3 Child and Family Research Institute, British Columbia Children’s Hospital, University of British Columbia, Vancouver, British Columbia, Canada; 4 Pediatrics and Environmental Health, Cincinnati Children’s Hospital Medical Center, Cincinnati, Ohio; 5 School of Dentistry, Indiana University, Indianapolis; 6 Department of Social and Preventive Medicine, Laval University, Québec City, Québec, Canada; 7 Centre de Recherche du CHU de Québec, Université Laval, Québec City, Québec, Canada; 8 School of Psychology, Laval University, Québec City, Québec, Canada

## Abstract

**Importance:**

The potential neurotoxicity associated with exposure to fluoride, which has generated controversy about community water fluoridation, remains unclear.

**Objective:**

To examine the association between fluoride exposure during pregnancy and IQ scores in a prospective birth cohort.

**Design, Setting, and Participants:**

This prospective, multicenter birth cohort study used information from the Maternal-Infant Research on Environmental Chemicals cohort. Children were born between 2008 and 2012; 41% lived in communities supplied with fluoridated municipal water. The study sample included 601 mother-child pairs recruited from 6 major cities in Canada; children were between ages 3 and 4 years at testing. Data were analyzed between March 2017 and January 2019.

**Exposures:**

Maternal urinary fluoride (MUF_SG_), adjusted for specific gravity and averaged across 3 trimesters available for 512 pregnant women, as well as self-reported maternal daily fluoride intake from water and beverage consumption available for 400 pregnant women.

**Main Outcomes and Measures:**

Children’s IQ was assessed at ages 3 to 4 years using the Wechsler Primary and Preschool Scale of Intelligence-III. Multiple linear regression analyses were used to examine covariate-adjusted associations between each fluoride exposure measure and IQ score.

**Results:**

Of 512 mother-child pairs, the mean (SD) age for enrollment for mothers was 32.3 (5.1) years, 463 (90%) were white, and 264 children (52%) were female. Data on MUF_SG_ concentrations, IQ scores, and complete covariates were available for 512 mother-child pairs; data on maternal fluoride intake and children’s IQ were available for 400 of 601 mother-child pairs. Women living in areas with fluoridated tap water (n = 141) compared with nonfluoridated water (n = 228) had significantly higher mean (SD) MUF_SG_ concentrations (0.69 [0.42] mg/L vs 0.40 [0.27] mg/L; *P* = .001; to convert to millimoles per liter, multiply by 0.05263) and fluoride intake levels (0.93 [0.43] vs 0.30 [0.26] mg of fluoride per day; *P* = .001). Children had mean (SD) Full Scale IQ scores of 107.16 (13.26), range 52-143, with girls showing significantly higher mean (SD) scores than boys: 109.56 (11.96) vs 104.61 (14.09); *P* = .001. There was a significant interaction (*P* = .02) between child sex and MUF_SG_ (6.89; 95% CI, 0.96-12.82) indicating a differential association between boys and girls. A 1-mg/L increase in MUF_SG_ was associated with a 4.49-point lower IQ score (95% CI, −8.38 to −0.60) in boys, but there was no statistically significant association with IQ scores in girls (*B* = 2.40; 95% CI, −2.53 to 7.33). A 1-mg higher daily intake of fluoride among pregnant women was associated with a 3.66 lower IQ score (95% CI, −7.16 to −0.14) in boys and girls.

**Conclusions and Relevance:**

In this study, maternal exposure to higher levels of fluoride during pregnancy was associated with lower IQ scores in children aged 3 to 4 years. These findings indicate the possible need to reduce fluoride intake during pregnancy.

## Key Points

**Question:**

Is maternal fluoride exposure during pregnancy associated with childhood IQ in a Canadian cohort receiving optimally fluoridated water?

**Findings:**

In this prospective birth cohort study, fluoride exposure during pregnancy was associated with lower IQ scores in children aged 3 to 4 years.

**Meaning:**

Fluoride exposure during pregnancy may be associated with adverse effects on child intellectual development, indicating the possible need to reduce fluoride intake during pregnancy.

## Teaser

This study examines the association between fluoride exposure during pregnancy and IQ scores in a prospective birth cohort.

## Introduction

For decades, community water fluoridation has been used to prevent tooth decay. Water fluoridation is supplied to about 66% of US residents, 38% of Canadian residents, and 3% of European residents.^1^ In fluoridated communities, fluoride from water and beverages made with tap water makes up 60% to 80% of daily fluoride intake in adolescents and adults.^2^

Fluoride crosses the placenta,^3^ and laboratory studies show that it accumulates in brain regions involved in learning and memory^4^ and alters proteins and neurotransmitters in the central nervous system.^5^ Higher fluoride exposure from drinking water has been associated with lower children’s intelligence in a meta-analysis^6^ of 27 epidemiologic studies and in studies^7,8^ including biomarkers of fluoride exposure. However, most prior studies were cross-sectional and conducted in regions with higher water fluoride concentrations (0.88-31.6 mg/L; to convert to millimoles per liter, multiply by 0.05263) than levels considered optimal (ie, 0.7 mg/L) in North America.^9^ Further, most studies did not measure exposure during fetal brain development. In a longitudinal birth cohort study involving 299 mother-child pairs in Mexico City, Mexico, a 1-mg/L increase in maternal urinary fluoride (MUF) concentration was associated with a 6-point (95% CI, −10.84 to −1.74) lower IQ score among school-aged children.^10^ In this same cohort, MUF was also associated with more attention-deficit/hyperactivity disorder–like symptoms.^11^ Urinary fluoride concentrations among pregnant women living in fluoridated communities in Canada are similar to concentrations among pregnant women living in Mexico City.^12^ However, it is unclear whether fluoride exposure during pregnancy is associated with cognitive deficits in a population receiving optimally fluoridated water.

This study examined whether exposure to fluoride during pregnancy was associated with IQ scores in children in a Canadian birth cohort in which 40% of the sample was supplied with fluoridated municipal water.

## Methods

### Study Cohort

Between 2008 and 2011, the Maternal-Infant Research on Environmental Chemicals (MIREC) program recruited 2001 pregnant women from 10 cities across Canada. Women who could communicate in English or French, were older than 18 years, and were within the first 14 weeks of pregnancy were recruited from prenatal clinics. Participants were not recruited if there was a known fetal abnormality, if they had any medical complications, or if there was illicit drug use during pregnancy. Additional details are in the cohort profile description.^13^

**Figure 1.**
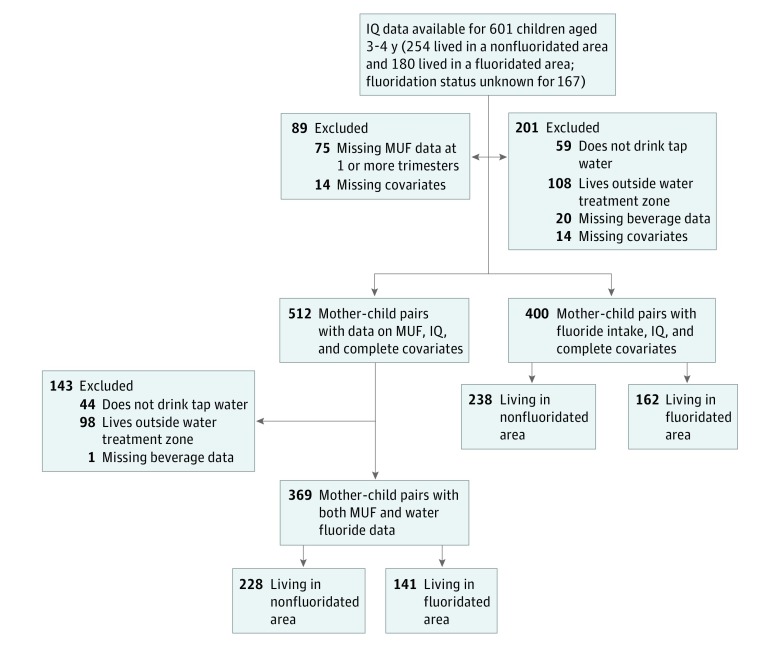
Flowchart of Inclusion Criteria MUF indicates maternal urinary fluoride.

A subset of 610 children in the MIREC Study was evaluated for the developmental phase of the study at ages 3 to 4 years; these children were recruited from 6 of 10 cities included in the original cohort: Vancouver, Montreal, Kingston, Toronto, Hamilton, and Halifax. Owing to budgetary restraints, recruitment was restricted to the 6 cities with the most participants who fell into the age range required for the testing during the data collection period. Of the 610 children, 601 (98.5%) completed neurodevelopmental testing; 254 (42.3%) of these children lived in nonfluoridated regions and 180 (30%) lived in fluoridated regions; for 167 (27.7%) fluoridation status was unknown owing to missing water data or reported not drinking tap water (Figure 1).

This study was approved by the research ethics boards at Health Canada, York University, and Indiana University. All women signed informed consent forms for both mothers and children.

### Maternal Urinary Fluoride Concentration

We used the mean concentrations of MUF measured in urine spot samples collected across each trimester of pregnancy at a mean (SD) of 11.57 (1.57), 19.11 (2.39), and 33.11 (1.50) weeks of gestation. Owing to the variability of urinary fluoride measurement and fluoride absorption during pregnancy,^14^ we only included women who had all 3 urine samples. In our previous work, these samples were moderately correlated; intraclass correlation coefficient (ICC) ranged from 0.37 to 0.40.^12^

Urinary fluoride concentration was analyzed at the Indiana University School of Dentistry using a modification of the hexamethyldisiloxane (Sigma Chemical Co) microdiffusion procedure^15^ and described in our previous work.^12^ Fluoride concentration could be measured to 0.02 mg/L. We excluded 2 samples (0.002%) because the readings exceeded the highest concentration standard (5 mg/L) and there was less certainty of these being representative exposure values.

To account for variations in urine dilution at the time of measurement, we adjusted MUF concentrations for specific gravity (SG) using the following equation: MUF_SG_ = MUF_i _× (SG_M_-1)/(SG_i_-1), where MUF_SG_ is the SG-adjusted fluoride concentration (in milligrams of fluoride per liter), MUF_i_ is the observed fluoride concentration, SG_i_ is the SG of the individual urine sample, and SG_M_ is the median SG for the cohort.^16^ For comparison, we also adjusted MUF using the same creatinine adjustment method that was used in the 2017 Mexican cohort.^10^

### Water Fluoride Concentration

Water treatment plants measured fluoride levels daily if fluoride was added to municipal drinking water and weekly or monthly if fluoride was not added to water.^12^ We matched participants’ postal codes with water treatment plant zones, allowing an estimation of water fluoride concentration for each woman by averaging water fluoride concentrations (in milligrams per liter) during the duration of pregnancy. We only included women who reported drinking tap water during pregnancy.

### Daily Fluoride Intake in Mothers

We obtained information on consumption of tap water and other water-based beverages (tea and coffee) from a self-report questionnaire completed by mothers during the first and third trimesters. This questionnaire was used in the original MREC cohort and has not been validated. Also, for this study, we developed methods to estimate and calculate fluoride intake that have not yet been validated. To estimate fluoride intake from tap water consumed per day (milligrams per day), we multiplied each woman’s consumption of water and beverages by her water fluoride concentration (averaged across pregnancy) and multiplied by 0.2 (fluoride content for a 200-mL cup). Because black tea contains a high fluoride content (2.6 mg/L),^17,18^ we also estimated the amount of fluoride consumed from black tea by multiplying each cup of black tea by 0.52 mg (mean fluoride content in a 200-mL cup of black tea made with deionized water) and added this to the fluoride intake variable. Green tea also contains varying levels of fluoride; therefore, we used the mean for the green teas listed by the US Department of Agriculture (1.935 mg/L).^18^ We multiplied each cup of green tea by 0.387 mg (fluoride content in a 200-mL cup of green tea made with deionized water) and added this to the fluoride intake variable.

### Primary Outcomes

We assessed children’s intellectual abilities with the Wechsler Preschool and Primary Scale of Intelligence, Third Edition. Full Scale IQ (FSIQ), a measure of global intellectual functioning, was the primary outcome. We also assessed verbal IQ (VIQ), representing verbal reasoning and comprehension, and performance IQ (PIQ), representing nonverbal reasoning, spatial processing, and visual-motor skills.

### Covariates

We selected covariates from a set of established factors associated with fluoride metabolism (eg, time of void and time since last void) and children’s intellectual abilities (eg, child sex, maternal age, gestational age, and parity) (Table 1). Mother’s race/ethnicity was coded as white or other, and maternal education was coded as either bachelor’s degree or higher or trade school diploma or lower. The quality of a child’s home environment was measured by the Home Observation for Measurement of the Environment (HOME)–Revised Edition^19^ on a continuous scale. We also controlled for city and, in some models, included self-reported exposure to secondhand smoke (yes/no) as a covariate.

**Table 1.** Demographic Characteristics and Exposure Outcomes for Mother-Child Pairs With MUF_SG_ (n = 512) and Fluoride Intake Data (n = 400) by Fluoridated and Nonfluoridated Status^a^

Variable^b^	No. (%)		
	MUF_SG_ Sample (n = 512)^c^	Maternal-Child Pairs With Fluoride Intake, IQ, and Complete Covariate Data (n = 400)	
		Nonfluoridated (n = 238)	Fluoridated (n = 162)
Mothers			
Age of mother at enrollment, mean (SD), y	32.33 (5.07)	32.61 (4.90)	32.52 (4.03)
Prepregnancy BMI, mean (SD)	25.19 (6.02)	25.19 (6.35)	24.33 (5.10)
Married or common law	497 (97)	225 (95)	159 (98)
Born in Canada	426 (83)	187 (79)	131 (81)
White	463 (90)	209 (88)	146 (90)
Maternal education			
Trade school diploma/high school	162 (32)	80 (34)	38 (24)
Bachelor’s degree or higher	350 (68)	158 (66)	124 (76)
Employed at time of pregnancy	452 (88)	205 (86)	149 (92)
Net income household >$70 000 CAD	364 (71)	162 (68)	115 (71)
HOME total score, mean (SD)	47.32 (4.32)	47.28 (4.48)	48.14 (3.90)
Smoked in trimester 1	12 (2)	7 (3)	2 (1)
Secondhand smoke in the home	18 (4)	9 (4)	2 (1)
Alcohol consumption, alcoholic drink/mo			
None	425 (83)	192 (81)	136 (84)
<1	41 (8)	23 (10)	11 (7)
≥1	46 (9)	23 (10)	15 (9)
Parity (first birth)	233 (46)	119 (50)	71 (44)
Children			
Female	264 (52)	118 (50)	83 (51)
Age at testing, mean (SD), y	3.42 (0.32)	3.36 (0.31)	3.49 (0.29)
Gestation, mean (SD), wk	39.12 (1.57)	39.19 (1.47)	39.17 (1.81)
Birth weight, mean (SD), kg	3.47 (0.49)	3.48 (0.48)	3.47 (0.53)
FSIQ	107.16 (13.26)	108.07 (13.31)	108.21 (13.72)
Boys^d^	104.61 (14.09)	106.31 (13.60)	104.78 (14.71)
Girls^d^	109.56 (11.96)	109.86 (12.83)	111.47 (11.89)
Exposure variables			
MUF_SG_ concentration, mg/L^e^			
No.	512	228	141
Mean (SD)	0.51 (0.36)	0.40 (0.27)	0.69 (0.42)
Fluoride intake level per day, mg			
No.	369^a^	238	162
Mean (SD)	0.54 (0.44)	0.30 (0.26)	0.93 (0.43)
Water fluoride concentration, mg/L			
No.	369^a^	238	162
Mean (SD)	0.31 (0.23)	0.13 (0.06)	0.59 (0.08)

Abbreviations: BMI, body mass index (calculated as weight in kilograms divided by height in meters squared); CAD, Canadian dollars; FSIQ, Full Scale IQ; HOME, Home Observation for Measurement of the Environment; MUF_SG_, maternal urinary fluoride adjusted for specific gravity.

SI conversion factor: To convert fluoride to millimoles per liter, multiply by 0.05263.

^a^ Owing to missing water treatment plant data and/or MUF data, the samples are distinct with some overlapping participants in both groups (n = 369).

^b^ All of the listed variables were tested as potential covariates, as well as the following: paternal variables (age, education, employment status, smoking status, and race/ethnicity); maternal chronic condition during pregnancy and birth country; breastfeeding duration; and time of void and time since last void.

^c^ Maternal urinary fluoride (averaged across all 3 trimesters) and corrected for specific gravity.

^d^ The FSIQ score has a mean (SD) of 100 (15); US population norms used.

^e^ Owing to missing water treatment plant data, the samples in the fluoridated and nonfluoridated regions do not add up to the MUF sample size.

### Statistical Analyses

In our primary analysis, we used linear regression analyses to estimate the associations between our 2 measures of fluoride exposure (MUF_SG_ and fluoride intake) and children’s FSIQ scores. In addition to providing the coefficient corresponding to a 1-mg difference in fluoride exposure, we also estimated coefficients corresponding to a fluoride exposure difference spanning the 25th to 75th percentile range (which corresponds to a 0.33 mg/L and 0.62 mg F/d difference in MUF_SG_ and fluoride intake, respectively) as well as the 10th to 90th percentile range (which corresponds to a 0.70 mg/L and 1.04 mg F/d difference in MUF_SG_ and fluoride intake, respectively).

We retained a covariate in the model if its *P* value was less than .20 or its inclusion changed the regression coefficient of the variable associated factor by more than 10% in any of the IQ models. Regression diagnostics confirmed that there were no collinearity issues in any of the IQ models with MUF_SG_ or fluoride intake (variance inflation factor <2 for all covariates). Residuals from each model had approximately normal distributions, and their Q-Q plots revealed no extreme outliers. Plots of residuals against fitted values did not suggest any assumption violations and there were no substantial influential observations as measured by Cook distance. Including quadratic or natural-log effects of MUF_SG_ or fluoride intake did not significantly improve the regression models. Thus, we present the more easily interpreted estimates from linear regression models. Additionally, we examined separate models with 2 linear splines to test whether the MUF_SG_ association significantly differed between lower and higher levels of MUF_SG_ based on 3 knots, which were set at 0.5 mg/L (mean MUF_SG_), 0.8 mg/L (threshold seen in the Mexican birth cohort),^10^ and 1 mg/L (optimal concentration in the United States until 2015).^20^ For fluoride intake, knots were set at 0.4 mg (mean fluoride intake), 0.8 mg, and 1 mg (in accordance with MUF_SG_). We also examined sex-specific associations in all models by testing the interactions between child sex and each fluoride measure.

In sensitivity analyses, we tested whether the associations between MUF_SG_ and IQ were confounded by maternal blood concentrations of lead,^21^ mercury,^21^ manganese,^21,22^ perfluoro-octanoic acid,^23^ or urinary arsenic.^24^ We also conducted sensitivity analyses by removing IQ scores that were greater than or less than 2.5 standard deviations from the sample mean. Additionally, we examined whether using MUF adjusted for creatinine instead of SG affected the results.

In additional analyses, we examined the association between our 2 measures of fluoride exposure (MUF_SG_ and fluoride intake) with VIQ and PIQ. Additionally, we examined whether water fluoride concentration was associated with FSIQ, VIQ, and PIQ scores.

For all analyses, statistical significance tests with a type I error rate of 5% were used to test sex interactions, while 95% confidence intervals were used to estimate uncertainty. Analyses were conducted using R software (the R Foundation).^25^ The *P *value level of significance was .05, and all tests were 2-sided.

## Results

For the first measure of fluoride exposure, MUF_SG_, 512 of 601 mother-child pairs (85.2%) who completed the neurodevelopmental visit had urinary fluoride levels measured at each trimester of the mother’s pregnancy and complete covariate data (Figure 1); 89 (14.8%) were excluded for missing MUF_SG_ at 1 or more trimesters (n = 75) or missing 1 or more covariates included in the regression (n = 14) (Figure 1). Of the 512 mother-child pairs with MUF_SG_ data (and all covariates), 264 children were female (52%).

For the second measure of fluoride exposure, fluoride intake from maternal questionnaire, data were available for 400 of the original 601 mother-child pairs (66.6%): 201 women (33.4%) were excluded for reporting not drinking tap water (n = 59), living outside of the predefined water treatment plant zone (n = 108), missing beverage consumption data (n = 20), or missing covariate data (n = 14) (Figure 1).

Children had mean FSIQ scores in the average range (population normed) (mean [SD], 107.16 [13.26], range = 52-143), with girls (109.56 [11.96]) showing significantly higher scores than boys (104.61 [14.09]; *P* < .001) (Table 1). The demographic characteristics of the 512 mother-child pairs included in the primary analysis were not substantially different from the original MIREC cohort or subset of mother-child pairs without 3 urine samples (eTable 1 in the Supplement). Of the 400 mother-child pairs with fluoride intake data (and all covariates), 118 of 238 (50%) in the group living in a nonfluoridated region were female and 83 of 162 (51%) in the group living in a fluoridated region were female.

### Fluoride Measurements

**Figure 2.**
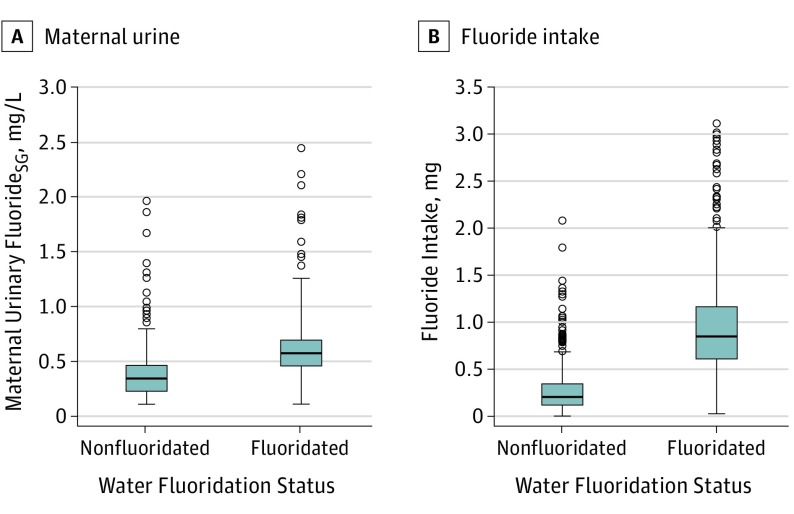
Distribution of Fluoride Levels in Maternal Urine and for Estimated Fluoride Intake by Fluoridation Status To convert fluoride to millimoles per liter, multiply by 0.05263.

The median MUF_SG_ concentration was 0.41 mg/L (range, 0.06-2.44 mg/L). Mean MUF_SG_ concentration was significantly higher among women (n = 141) who lived in communities with fluoridated drinking water (0.69 [0.42] mg/L) compared with women (n=228) who lived in communities without fluoridated drinking water (0.40 [0.27] mg/L; *P* < .001) (Table 1; Figure 2).

The median estimated fluoride intake was 0.39 mg per day (range, 0.01-2.65 mg). As expected, the mean (SD) fluoride intake was significantly higher for women (162 [40.5%]) who lived in communities with fluoridated drinking water (mean [SD], 0.93 [0.43] mg) than women (238 [59.5%]) who lived in communities without fluoridated drinking water (0.30 [0.26] mg; *P* < .001) (Table 1; Figure 2). The MUF_SG_ was moderately correlated with fluoride intake (*r* = 0.49; *P* < .001) and water fluoride concentration (*r* = 0.37; *P* < .001).

### Maternal Urinary Fluoride Concentrations and IQ

Before covariate adjustment, a significant interaction (*P* for interaction = .03) between MUF_SG_ and child sex (*B *= 7.24; 95% CI, 0.81- 13.67) indicated that MUF_SG_ was associated with FSIQ in boys; an increase of 1 mg/L MUF_SG_ was associated with a 5.01 (95% CI, −9.06 to −0.97; *P* = .02) lower FSIQ score in boys. In contrast, MUF_SG_ was not significantly associated with FSIQ score in girls (*B *= 2.23; 95% CI, −2.77 to 7.23; *P = .*38) (Table 2).

**Table 2.** Unadjusted and Adjusted Associations Estimated From Linear Regression Models of Fluoride Exposure Variables and FSIQ Scores

Variable	Difference (95% CI)			
	Unadjusted	Adjusted Estimates, Regression Coefficients Indicate Change in Outcome per^a^		
		1 mg	25th to 75th Percentiles	10th to 90th Percentiles
MUF_SG_^b^^,^^c^	−2.60 (−5.80 to 0.60)	−1.95 (−5.19 to 1.28)	−0.64 (−1.69 to 0.42)	−1.36 (−3.58 to 0.90)
Boys	−5.01 (−9.06 to −0.97)	−4.49 (−8.38 to −0.60)	−1.48 (−2.76 to −0.19)	−3.14 (−5.86 to −0.42)
Girls	2.23 (−2.77 to 7.23)	2.40 (−2.53 to 7.33)	0.79 (−0.83 to 2.42)	1.68 (−1.77 to 5.13)
Fluoride intake^d^^,^^e^	−3.19 (−5.94 to −0.44)	−3.66 (−7.16 to −0.15)	−2.26 (−4.45 to −0.09)	−3.80 (−7.46 to −0.16)

Abbreviations: FSIQ, Full Scale IQ; HOME, Home Observation for Measurement of the Environment; MUF_SG_, maternal urinary fluoride adjusted for specific gravity.

^a^ Adjusted estimates pertain to predicted FSIQ difference for a value spanning the interquartile range (25th to 75th percentiles) and 80th central range (10th to 90th percentiles): (1) MUF_SG_: 0.33 mg/L, 0.70 mg/L, respectively; (2) fluoride intake: 0.62 mg, 1.04 mg, respectively.

^b^ n = 512.

^c^ Adjusted for city, HOME score, maternal education, race/ethnicity, and including child sex interaction.

^d^ n = 400.

^e^ Adjusted for city, HOME score, maternal education, race/ethnicity, child sex, and prenatal secondhand smoke exposure.

**Figure 3.**
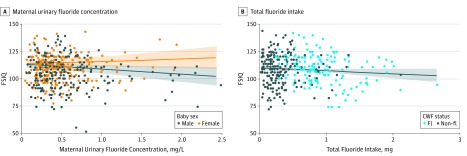
Covariate Results of Multiple Linear Regression Models of Full Scale IQ (FSIQ) from Maternal Urinary Fluoride Concentration by Child Sex (n = 512) and Total Fluoride Intake Estimated from Daily Maternal Beverage Consumption (n = 400) B, Community fluoridation status (CWF) is shown for each woman; black dots represent women living in nonfluoridated (non-Fl) communities and blue dots represent women living in fluoridated (Fl) communities.

Adjusting for covariates, a significant interaction (*P* for interaction = .02) between child sex and MUF_SG_ (*B *= 6.89; 95% CI, 0.96-12.82) indicated that an increase of 1 mg/L of MUF_SG_ was associated with a 4.49 (95% CI, −8.38 to −0.60; *P *= .02) lower FSIQ score for boys. An increase from the 10th to 90th percentile of MUF_SG_ was associated with a 3.14 IQ decrement among boys (Table 2; Figure 3). In contrast, MUF_SG_ was not significantly associated with FSIQ score in girls (*B = *2.43; 95% CI, −2.51 to 7.36; *P* = .33).

### Estimated Fluoride Intake and IQ

A 1-mg increase in fluoride intake was associated with a 3.66 (95% CI, −7.16 to −0.15; *P* = .04) lower FSIQ score among boys and girls (Table 2; Figure 3). The interaction between child sex and fluoride intake was not statistically significant *(B = *1.17; 95% CI, −4.08 to 6.41; *P* for interaction = .66).

### Sensitivity Analyses

Adjusting for lead, mercury, manganese, perfluorooctanoic acid, or arsenic concentrations did not substantially change the overall estimates of MUF_SG_ for boys or girls (eTable 2 in the Supplement). Use of MUF adjusted for creatinine did not substantially alter the associations with FSIQ (eTable 2 in the Supplement). Including time of void and time since last void did not substantially change the regression coefficient of MUF_SG_ among boys or girls.

Estimates for determining the association between MUF_SG_ and PIQ showed a similar pattern with a statistically significant interaction between MUF_SG _and child sex (*P *for interaction = .007). An increase of 1 mg/L MUF_SG_ was associated with a 4.63 (95% CI, −9.01 to −0.25; *P* = .04) lower PIQ score in boys, but the association was not statistically significant in girls (*B = *4.51; 95% CI, −1.02 to 10.05; *P* = .11). An increase of 1 mg/L MUF_SG_ was not significantly associated with VIQ in boys (*B *= −2.85; 95% CI, −6.65 to 0.95; *P* = .14) or girls (*B *= 0.55; 95% CI, −4.28 to 5.37; *P* = .82); the interaction between MUF_SG_ and child sex was not statistically significant (*P *for interaction = .25) (eTable 3 in the Supplement).

 Consistent with the findings on estimated maternal fluoride intake, increased water fluoride concentration (per 1 mg/L) was associated with a 5.29 (95% CI, −10.39 to −0.19) lower FSIQ score among boys and girls and a 13.79 (95% CI, −18.82 to −7.28) lower PIQ score (eTable 4 in the Supplement).

## Discussion

Using a prospective Canadian birth cohort, we found that estimated maternal exposure to higher fluoride levels during pregnancy was associated with lower IQ scores in children. This association was supported by converging findings from 2 measures of fluoride exposure during pregnancy. A difference in MUF_SG_ spanning the interquartile range for the entire sample (ie, 0.33 mg/L), which is roughly the difference in MUF_SG_ concentration for pregnant women living in a fluoridated vs a nonfluoridated community, was associated with a 1.5-point IQ decrement among boys. An increment of 0.70 mg/L in MUF_SG_ concentration was associated with a 3-point IQ decrement in boys; about half of the women living in a fluoridated community have a MUF_SG_ equal to or greater than 0.70 mg/L. These results did not change appreciably after controlling for other key exposures such as lead, arsenic, and mercury.

To our knowledge, this study is the first to estimate fluoride exposure in a large birth cohort receiving optimally fluoridated water. These findings are consistent with that of a Mexican birth cohort study that reported a 6.3 decrement in IQ in preschool-aged children compared with a 4.5 decrement for boys in our study for every 1 mg/L of MUF.^10^ The findings of the current study are also concordant with ecologic studies that have shown an association between higher levels of fluoride exposure and lower intellectual abilities in children.^7,8,26^ Collectively, these findings support that fluoride exposure during pregnancy may be associated with neurocognitive deficits.

In contrast with the Mexican study,^10^ the association between higher MUF_SG_ concentrations and lower IQ scores was observed only in boys but not in girls. Studies of fetal and early childhood fluoride exposure and IQ have rarely examined differences by sex; of those that did, some reported no differences by sex.^10,27,28,29^ Most rat studies have focused on fluoride exposure in male rats,^30^ although 1 study^31^ showed that male rats were more sensitive to neurocognitive effects of fetal exposure to fluoride. Testing whether boys are potentially more vulnerable to neurocognitive effects associated with fluoride exposure requires further investigation, especially considering that boys have a higher prevalence of neurodevelopmental disorders such as ADHD, learning disabilities, and intellectual disabilities.^32^ Adverse effects of early exposure to fluoride may manifest differently for girls and boys, as shown with other neurotoxicants.^33,34,35,36^

The estimate of maternal fluoride intake during pregnancy in this study showed that an increase of 1 mg of fluoride was associated with a decrease of 3.7 IQ points across boys and girls. The finding observed for fluoride intake in both boys and girls may reflect postnatal exposure to fluoride, whereas MUF primarily captures prenatal exposure. Importantly, we excluded women who reported that they did not drink tap water and matched water fluoride measurements to time of pregnancy when estimating maternal fluoride intake. None of the fluoride concentrations measured in municipal drinking water were greater than the maximum acceptable concentration of 1.5 mg/L set by Health Canada; most (94.3%) were lower than the 0.7 mg/L level considered optimal.^37^

Water fluoridation was introduced in the 1950s to prevent dental caries before the widespread use of fluoridated dental products. Originally, the US Public Health Service set the optimal fluoride concentrations in water from 0.7 to 1.2 mg/L to achieve the maximum reduction in tooth decay and minimize the risk of enamel fluorosis.^38^ Fluorosis, or mottling, is a symptom of excess fluoride intake from any source occurring during the period of tooth development. In 2012, 68% of adolescents had very mild to severe enamel fluorosis.^39^ The higher prevalence of enamel fluorosis, especially in fluoridated areas,^40^ triggered renewed concern about excessive ingestion of fluoride. In 2015, in response to fluoride overexposure and rising rates of enamel fluorosis,^39,41,42^ the US Public Health Service recommended an optimal fluoride concentration of 0.7 mg/L, in line with the recommended level of fluoride added to drinking water in Canada to prevent caries. However, the beneficial effects of fluoride predominantly occur at the tooth surface after the teeth have erupted.^43^ Therefore, there is no benefit of systemic exposure to fluoride during pregnancy for the prevention of caries in offspring.^44^ The evidence showing an association between fluoride exposure and lower IQ scores raises a possible new concern about cumulative exposures to fluoride during pregnancy, even among pregnant women exposed to optimally fluoridated water.

### Strengths and Limitations

Our study has several strengths and limitations. First, urinary fluoride has a short half-life (approximately 5 hours) and depends on behaviors that were not controlled in our study, such as consumption of fluoride-free bottled water or swallowing toothpaste prior to urine sampling. We minimized this limitation by using 3 serial urine samples and tested for time of urine sample collection and time since last void, but these variables did not alter our results. Second, although higher maternal ingestion of fluoride corresponds to higher fetal plasma fluoride levels,^45^ even serial maternal urinary spot samples may not precisely represent fetal exposure throughout pregnancy. Third, while our analyses controlled for a comprehensive set of covariates, we did not have maternal IQ data. However, there is no evidence suggesting that fluoride exposure differs as a function of maternal IQ; our prior study did not observe a significant association between MUF levels and maternal education level.^12^ Moreover, a greater proportion of women living in fluoridated communities (124 [76%]) had a university-level degree compared with women living in nonfluoridated communities (158 [66%]). Nonetheless, despite our comprehensive array of covariates included, this observational study design could not address the possibility of other unmeasured residual confounding. Fourth, fluoride intake did not measure actual fluoride concentration in tap water in the participant’s home; Toronto, for example, has overlapping water treatment plants servicing the same household. Similarly, our fluoride intake estimate only considered fluoride from beverages; it did not include fluoride from other sources such as dental products or food. Furthermore, fluoride intake data were limited by self-report of mothers’ recall of beverage consumption per day, which was sampled at 2 points of pregnancy, and we lacked information regarding specific tea brand.^17,18^ In addition, our methods of estimating maternal fluoride intake have not been validated; however, we show construct validity with MUF. Fifth, this study did not include assessment of postnatal fluoride exposure or consumption. However, our future analyses will assess exposure to fluoride in the MIREC cohort in infancy and early childhood.

## Conclusions

In this prospective birth cohort study from 6 cities in Canada, higher levels of fluoride exposure during pregnancy were associated with lower IQ scores in children measured at age 3 to 4 years. These findings were observed at fluoride levels typically found in white North American women. This indicates the possible need to reduce fluoride intake during pregnancy.

## Notes

Supplement. **eTable 1.** Comparison of Current Sample to Other MIREC Samples **eTable 2.** Sensitivity Analyses Predicting Full Scale IQ (FSIQ) **eTable 3.** Unadjusted and Adjusted Effect Estimates From Linear Regression Models Of Fluoride Exposure Variables Predicting Verbal IQ and Performance IQ Scores **eTable 4.** Unadjusted and Adjusted Effect Estimates From Linear Regression Models of Water Fluoride Concentration (mg/L) Predicting FSIQ Scores

## References

[1] Public Health Agency of Canada The state of Community Water Fluoridation (CWF) across Canada. https://www.canada.ca/en/services/health/publications/healthy-living/community-water-fluoridation-across-canada-2017.html. Accessed June 15, 2018.

[2] United States Environmental Protection Agency Fluoride: Relative Source Contribution Analysis. Vol 820-R-10-0. https://www.epa.gov/sites/production/files/2019-03/documents/comment-response-report-peer-review-fluoride-exposure.pdf. Published 2010. Accessed May 18, 2017.

[3] Ron M, Singer L, Menczel J, Kidroni G Fluoride concentration in amniotic fluid and fetal cord and maternal plasma. Eur J Obstet Gynecol Reprod Biol. 1986;21(4):213-218. doi:10.1016/0028-2243(86)90018-3 3709921

[4] Pereira M, Dombrowski PA, Losso EM, Chioca LR, Da Cunha C, Andreatini R Memory impairment induced by sodium fluoride is associated with changes in brain monoamine levels. Neurotox Res. 2011;19(1):55-62. doi:10.1007/s12640-009-9139-5 19957215

[5] Jiang C, Zhang S, Liu H, Low glucose utilization and neurodegenerative changes caused by sodium fluoride exposure in rat’s developmental brain. Neuromolecular Med. 2014;16(1):94-105. doi:10.1007/s12017-013-8260-z 23982469

[6] Choi AL, Sun G, Zhang Y, Grandjean P Developmental fluoride neurotoxicity: a systematic review and meta-analysis. Environ Health Perspect. 2012;120(10):1362-1368. doi:10.1289/ehp.1104912 22820538 PMC3491930

[7] Das K, Mondal NK Dental fluorosis and urinary fluoride concentration as a reflection of fluoride exposure and its impact on IQ level and BMI of children of Laxmisagar, Simlapal Block of Bankura District, W.B., India. Environ Monit Assess. 2016;188(4):218. doi:10.1007/s10661-016-5219-1 26960765

[8] Valdez Jiménez L, López Guzmán OD, Cervantes Flores M, In utero exposure to fluoride and cognitive development delay in infants. Neurotoxicology. 2017;59:65-70. doi:10.1016/j.neuro.2016.12.011 28077305

[9] U.S. Department of Health and Human Services Federal Panel on Community Water Fluoridation U.S. public health service recommendation for fluoride concentration in drinking water for the prevention of dental caries. Public Health Rep. 2015;130(1):21-28. doi:10.1177/003335491513000408 26346489 PMC4547570

[10] Bashash M, Thomas D, Hu H, Prenatal fluoride exposure and cognitive outcomes in children at 4 and 6 – 12 years of age in Mexico. Enviromental Heal Perspect. 2017;1:1-12. 28937959 10.1289/EHP655 PMC5915186

[11] Bashash M, Marchand M, Hu H, Prenatal fluoride exposure and attention deficit hyperactivity disorder (ADHD) symptoms in children at 6-12 years of age in Mexico City. Environ Int. 2018;121(Pt 1):658-666. doi:10.1016/j.envint.2018.09.017 30316181

[12] Till C, Green R, Grundy JG, Community water fluoridation and urinary fluoride concentrations in a national sample of pregnant women in Canada. Environ Health Perspect. 2018;126(10):107001. doi:10.1289/EHP3546 30392399 PMC6371693

[13] Arbuckle TE, Fraser WD, Fisher M, Cohort profile: the maternal-infant research on environmental chemicals research platform. Paediatr Perinat Epidemiol. 2013;27(4):415-425. doi:10.1111/ppe.12061 23772943

[14] Opydo-Szymaczek J, Borysewicz-Lewicka M Urinary fluoride levels for assessment of fluoride exposure of pregnant women in Poznan, Poland. Fluoride. 2005;38(4):312-317.

[15] Martínez-Mier EA, Cury JA, Heilman JR, Development of gold standard ion-selective electrode-based methods for fluoride analysis. Caries Res. 2011;45(1):3-12. doi:10.1159/000321657 21160184 PMC3696354

[16] Macpherson S, Arbuckle TE, Fisher M Adjusting urinary chemical biomarkers for hydration status during pregnancy. J Expo Sci Environ Epidemiol. 2018;28:481-493. doi:10.1038/s41370-018-0043-z 29880833 PMC8075920

[17] Waugh DT, Potter W, Limeback H, Godfrey M Risk Assessment of fluoride intake from tea in the Republic of Ireland and its implications for public health and water fluoridation. Int J Environ Res Public Health. 2016;13(3):259. doi:10.3390/ijerph13030259 26927146 PMC4808922

[18] USDA Nutrient Data Laboratory Beltsville Human Nutrition Research Center Agricultural Research Service USDA National Fluoride Database of Selected Beverages and Foods. http://www.ars.usda.gov/SP2UserFiles/Place/80400525/Data/Fluoride/F02.pdf. Published 2005. Accessed May 18, 2017.

[19] Caldwell B, Bradley R *Home Observation for Measurement of the Environment (HOME): Revised Edition* Little Rock, Arkansas: University of Arkansas; 1984.

[20] Rabb-Waytowich D Water fluoridation in Canada: past and present. J Can Dent Assoc. 2009;75(6):451-454. 19627654

[21] Arbuckle TE, Liang CL, Morisset A-S, ; MIREC Study Group Maternal and fetal exposure to cadmium, lead, manganese and mercury: the MIREC study. Chemosphere. 2016;163:270-282. doi:10.1016/j.chemosphere.2016.08.023 27540762

[22] Dion L-A, Saint-Amour D, Sauvé S, Barbeau B, Mergler D, Bouchard MF Changes in water manganese levels and longitudinal assessment of intellectual function in children exposed through drinking water. Neurotoxicology. 2018;64:118-125. doi:10.1016/j.neuro.2017.08.015 28870865

[23] Vélez MP, Arbuckle TE, Fraser WD Maternal exposure to perfluorinated chemicals and reduced fecundity: the MIREC study. Hum Reprod. 2015;30(3):701-709. doi:10.1093/humrep/deu350 25567616 PMC4325673

[24] Ettinger AS, Arbuckle TE, Fisher M, ; MIREC Study Group Arsenic levels among pregnant women and newborns in Canada: results from the Maternal-Infant Research on Environmental Chemicals (MIREC) cohort. Environ Res. 2017;153:8-16. doi:10.1016/j.envres.2016.11.008 27880879

[25] Team RCR A Language and Environment for Statistical Computing. Vienna, Austria: R Foundation; 2013.

[26] Choi AL, Zhang Y, Sun G, Association of lifetime exposure to fluoride and cognitive functions in Chinese children: a pilot study. Neurotoxicol Teratol. 2015;47:96-101. doi:10.1016/j.ntt.2014.11.001 25446012

[27] Lu Y, Sun ZR, Wu LN, Wang X, Lu W, Liu SS Effect of high-fluoride water on intelligence in children. Fluoride. 2000;33(2):74-78.

[28] Zhao LB, Liang GH, Zhang DN, Wu XR Effect of high fluoride water supply on children’s intelligence. Fluoride. 1996;29(4):190-192.

[29] Xiang Q, Liang Y, Chen L, Effect of fluoride in drinking water on children’s intelligence. Fluoride. 2003;36(2):84-94.

[30] McPherson CA, Zhang G, Gilliam R, An evaluation of neurotoxicity following fluoride exposure from gestational through adult ages in Long-Evans hooded rats. Neurotox Res. 2018;34(4):781-798. doi:10.1007/s12640-018-9870-x 29404855 PMC6077107

[31] Mullenix PJ, Denbesten PK, Schunior A, Kernan WJ Neurotoxicity of sodium fluoride in rats. Neurotoxicol Teratol. 1995;17(2):169-177. doi:10.1016/0892-0362(94)00070-T 7760776

[32] Boyle CA, Boulet S, Schieve LA, Trends in the prevalence of developmental disabilities in US Children, 1997–2008. http://pediatrics.aappublications.org/content/early/2011/05/19/peds.2010-2989. Published 2011. Accessed May 30, 2017. 10.1542/peds.2010-2989 21606152

[33] Gochfeld M Sex differences in human and animal toxicology. Toxicol Pathol. 2017;45(1):172-189. doi:10.1177/0192623316677327 27895264 PMC5371029

[34] Arbuckle TE Are there sex and gender differences in acute exposure to chemicals in the same setting? Environ Res. 2006;101(2):195-204. doi:10.1016/j.envres.2005.08.015 16233896

[35] Desrochers-Couture M, Oulhote Y, Arbuckle TE, Prenatal, concurrent, and sex-specific associations between blood lead concentrations and IQ in preschool Canadian children. Environ Int. 2018;121(Pt 2):1235-1242. doi:10.1016/j.envint.2018.10.043 30392942

[36] Evans SF, Kobrosly RW, Barrett ES, Prenatal bisphenol A exposure and maternally reported behavior in boys and girls. Neurotoxicology. 2014;45:91-99. doi:10.1016/j.neuro.2014.10.003 25307304 PMC4362616

[37] Health Canada *Guidelines for Canadian Drinking Water Quality: Guideline Technical Document* Ottawa, Ontario: Ottawa, Ontario, Air and Climate Change Bureau, Healthy Environments and Consumer Safety Branch, Health Canada; 2010.

[38] Martinez-Mier EA, Shone DB, Buckley CM, Ando M, Lippert F, Soto-Rojas AE Relationship between enamel fluorosis severity and fluoride content. J Dent. 2016;46:42-46. doi:10.1016/j.jdent.2016.01.007 26808157 PMC4767679

[39] Wiener RC, Shen C, Findley P, Tan X, Sambamoorthi U Dental fluorosis over time: a comparison of national health and nutrition examination survey data from 2001-2002 and 2011-2012. J Dent Hyg. 2018;92(1):23-29. 29500282 PMC5929463

[40] National Research Council (NRC) Fluoride in drinking water: a scientific review of EPA’s standards. Washington, DC: National Academies Press; 2006.

[41] Beltrán-Aguilar ED, Barker L, Dye BA Prevalence and severity of dental fluorosis in the United States, 1999-2004. NCHS Data Brief. 2010;(53):1-8. 21211168

[42] Warren JJ, Kanellis MJ, Levy SM Fluorosis of the primary dentition: what does it mean for permanent teeth? J Am Dent Assoc. 1999;130(3):347-356. doi:10.14219/jada.archive.1999.0204 10085657

[43] Limeback H A re-examination of the pre-eruptive and post-eruptive mechanism of the anti-caries effects of fluoride: is there any anti-caries benefit from swallowing fluoride? Community Dent Oral Epidemiol. 1999;27(1):62-71. doi:10.1111/j.1600-0528.1999.tb01993.x 10086928

[44] Takahashi R, Ota E, Hoshi K, Fluoride supplementation (with tablets, drops, lozenges or chewing gum) in pregnant women for preventing dental caries in the primary teeth of their children. Cochrane Database Syst Rev. 2017;10(10):CD011850. doi:10.1002/14651858.CD011850.pub2 29059464 PMC6485723

[45] Gedalia I, Zukerman H, Leventhal H Fluoride content of teeth and bones of human fetuses: in areas with about 1 ppm of fluoride in drinking water. J Am Dent Assoc. 1965;71(5):1121-1123. doi:10.14219/jada.archive.1965.0051 5213647

