# Supplementary material for: Association Between Maternal Fluoride Exposure During Pregnancy and IQ Scores in Offspring in Canada
Source: JAMA Pediatr. 2019 Aug 19;173(10):940–8. doi: 10.1001/jamapediatrics.2019.1729 (PMC6704756; doi:10.1001/jamapediatrics.2019.1729)
Supplement: Supplement. — eTable 1. Comparison of Current Sample to Other MIREC Samples eTable 2. Sensitivity Analyses Predicting Full Scale IQ (FSIQ) eTable 3. Unadjusted and Adjusted Effect Estimates From Linear Regression Models Of Fluoride Exposure Variables Predicting Verbal IQ and Performance IQ Scores eTable 4. Unadjusted and Adjusted Effect Estimates From Linear Regression Models of Water Fluoride Concentration (mg/L) Predicting FSIQ Scores [file jamapediatr-173-940-s001.pdf]

Supplementary Online Content

Green R, Lanphear B, Hornung R, et al. Association between maternal fluoride exposure during pregnancy and IQ scores in offspring in Canada. Published online August 19, 2019. *JAMA Pediatr.* doi:10.1001/jamapediatrics.2019.1729

eTable 1. Comparison of Current Sample to Other MIREC Samples

eTable 2. Sensitivity Analyses Predicting Full Scale IQ (FSIQ)

eTable 3. Unadjusted and Adjusted Effect Estimates From Linear Regression Models Of Fluoride Exposure Variables Predicting Verbal IQ and Performance IQ Scores

eTable 4. Unadjusted and Adjusted Effect Estimates From Linear Regression Models of Water Fluoride Concentration (mg/L) Predicting FSIQ Scores

This supplementary material has been provided by the authors to give readers additional information about their work.

© 2019 Green R et al. *JAMA Pediatr.*

eTable 1: Comparison of current sample to other MIREC samples

Variable	Participants in the MIREC cohort with:		
	Live births[a]	Women with 3 urine samples and child IQ scores	Women with <3 urine samples and child IQ scores
n	1983	512	70
Mean (SD) age of mother at enrollment (years)	32.2 (5.1)	32.51 (4.46)	32.43 (5.29)
Caucasian, No. (%)	1651 (85)	463 (90)	56 (80)
Married or Common-law, No. (%)	1890 (95.3)	497 (97)	63 (90)
Born in Canada, No. (%)	1569 (79)	426 (83)	53 (76)
Maternal Education, No. (%)			
High school or less	158 (9)	24 (5)	4 (6)
Some college	100 (5)	17 (3)	5 (7)
College diploma	412 (24)	121 (24)	20 (29)
University degree	1246 (62)	348 (68)	40 (57)
Employed at time of pregnancy, No. (%)	1647 (83)	452 (88)	87.0
Net household income >$70,000, No. (%)	1269 (64)	364 (71)	65.2

Abbreviations: SD = standard deviation

[a]from a total of 2001 women who were recruited

Note: Differences between the analytic sample (n=512), live births sample (n=1983), and excluded participants (n=70) were all considered small (i.e. Cohen's effect size *h* of ≤0.30).

© 2019 Green R et al. *JAMA Pediatr.*

eTable 2: Sensitivity analyses predicting Full Scale IQ (FSIQ).

**MLR Models**	***N***	***B (SE)* of predictor**	***p***	**95% CI**
Model $_{A}$	512	-4.49 (1.98)	.02	-8.38, -0.60
Model $_{A+lead}$	504	-4.61 (1.98)	.02	-8.50, -0.71
Model $_{A+mercury}$	456	-5.13 (2.05)	.01	-9.16, -1.10
Model $_{A+PFOA}$	503	-4.57 (1.97)	.02	-8.21, -0.50
Model $_{A+arsenic}$	512	-4.44 (1.99)	.03	-8.35, -0.54
Model $_{A+manganese}$	502	-4.55 (1.97)	.02	-8.42, -0.69
Model $_{A+second\ hand\ smoke\ exposure}$	512	-4.18 (1.98)	.03	-8.06, -0.30
Model $_{B}$	510	-4.11 (1.92)	.03	-7.89, -0.33
Model $_{C}$	407	-4.96 (1.83)	.007	-8.56, -1.36
Model $_{D}$	369	-6.25 (2.70)	.02	-11.56, -0.94

Abbreviations: HOME = Home Observation for Measurement of the Environment; PFOA = perfluorooctanoic acid; MLR = multiple linear regression; MUF = maternal urinary fluoride

Model $_{A}$ – MUF_{SG} coefficient for boys controlling for city, HOME total score, race and maternal level of education with baby sex as an interaction term

Model $_{B}$ – Model $_{A}$ without two boys with FSIQ lower than 60

Model $_{C}$ – MUF coefficient for boys adjusted for creatinine with same covariates as Model $_{A}$

Model $_{D}$ – using water fluoride concentration as a predictor for those women who have MUF values only

© 2019 Green R et al. *JAMA Pediatr.*

eTable 3. Unadjusted and adjusted effect estimates from linear regression models of fluoride exposure variables predicting Verbal IQ and Performance IQ scores.

	Performance IQ		Verbal IQ	
	Unadjusted	Adjusted	Unadjusted	Adjusted
**Predictor**	***B* (95% CI)**	***B* (95% CI)**	***B* (95% CI)**	***B* (95% CI)**
MUF_{SG}[a]	-5.81* (-9.31, -2.30)	-1.24 (-4.88, 2.40)	1.28 (-1.87, 4.43)	-1.60 (-4.74, 1.55)
Boys	-8.81* (-13.29, -4.32)	-4.63* (-9.01, -0.25)[d]	-0.21 (-4.19, 3.77)	-2.82 (-6.62, 0.98)[c]
Girls[b]	-0.56 (-6.09, 4.97)	4.51 (-1.02, 10.05)[d]	4.78 (-0.14, 9.70)	0.50 (-4.32, 5.33)[c]
Fluoride intake[c]	-5.75* (-8.74, -2.76)	-2.74 (-6.82, 1.34)[e]	-0.03 (-2.71, 2.64)	-3.08 (-6.40, 0.25)[d]

Abbreviations: MUF_{SG} = maternal urinary fluoride adjusted for specific gravity; HOME = Home Observation for Measurement of the Environment

[a] N=507 for PIQ; N=509 for VIQ

[b] Girls had significantly higher scores on VIQ ($p < .001$) and PIQ ($p = .03$) compared with boys

[c] N=395 for PIQ; N=399 for VIQ; Missing data due to incomplete questionnaire responses to beverage consumption

[d] adjusted for city, HOME score, maternal education, race and including child sex interaction

[e] adjusted for HOME score, maternal education, race, child sex, prenatal second-hand smoke exposure, and city

*$p < .05$

© 2019 Green R et al. *JAMA Pediatr.*

eTable 4: Unadjusted and adjusted effect estimates from linear regression models of water fluoride concentration (mg/L) predicting FSIQ scores.

		Adjusted estimates[b]		
	Unadjusted (FSIQ)	Full Scale IQ	Performance IQ	Verbal IQ
**Predictor**	***B* (95% CI)**	***B* (95% CI)**	***B* (95% CI)**	***B* (95% CI)**
Water fluoride concentration[a]	3.49 (-9.04, 2.06)	-5.29* (-10.39, -0.19)[b]	-13.79* (-18.82, -7.28)	3.37 (-1.50, 8.24)

[a] N=420

[b] adjusted for HOME score, maternal education, race, child sex, and prenatal second-hand smoke exposure; because city was strongly multi-collinear with water fluoride concentration (VIF >20), it was excluded from the model

*$p < .05$

© 2019 Green R et al. *JAMA Pediatr.*
